# Orsellinic Acid from the Endophytic *Fusarium oxysporum* Drives Specialized Metabolism in *Peperomia obtusifolia*

**DOI:** 10.1021/acsomega.5c12178

**Published:** 2026-07-15

**Authors:** Wellington Gomes de Lima, Andreia de Araújo Morandim-Giannetti, João Luiz Bronzel Júnior, Silvia Noelí López, Massuo Jorge Kato, Maysa Furlan

**Affiliations:** † Institute of Chemistry, Universidade Estadual Paulista (UNESP), Araraquara, São Paulo 14800-900, Brazil; ‡ Department of Chemical Engineering, Centro Universitário da FEI, São Bernardo do Campo, São Paulo 09850-901, Brazil; § Pharmacognosy, Faculty of Biochemical and Pharmaceutical Sciences, Universidad Nacional de Rosario - CONICET, Rosario 2000, Argentina; ∥ Institute of Chemistry, Universidade de São Paulo (USP), São Paulo, São Paulo 05508-000, Brazil

## Abstract

The metabolic cooperation between plants and their endophytic fungi represents a promising frontier in the biosynthesis of natural products. This study elucidates the contribution of the endophytic fungus *Fusarium oxysporum* Po18 to the production of aromatic polyketides that drive specialized metabolism in *Peperomia obtusifolia*. Cultivation parameters for *F. oxysporum* were optimized using a Central Composite Rotatable Design (CCRD), revealing that mild temperatures (28 °C) and extended incubation (9 days) maximized orsellinic acid accumulation. LC–MS/MS identified orsellinic acid as [M–H]– at *m*/*z* 167.0356, with the diagnostic fragment ion *m*/*z* 122.8924, and quantified by HPLC–DAD, achieving a concentration of 132 μg/mL under optimized conditions. Comparative metabolomic analysis and molecular networking (GNPS) revealed related fungal metabolites, including lecanoric acid, 6-methylsalicylic acid, and citrinin, all derived from the fungal polyketide synthase (PKS) pathway. These metabolites are proposed to act as biosynthetic precursors for chroman and benzopyran derivatives previously reported in *P. obtusifolia*. The results provide the first experimental evidence of a biosynthetic partnership between *Fusarium* and *Peperomia*, in which the endophyte may supply aromatic scaffolds that could subsequently undergo downstream modifications in the host plant. This study expands the understanding of fungal–plant metabolic interactions and highlights *F. oxysporum* as a sustainable biotechnological source of aromatic polyketides with potential applications in natural product chemistry and biocatalysis.

## Graphical abstract

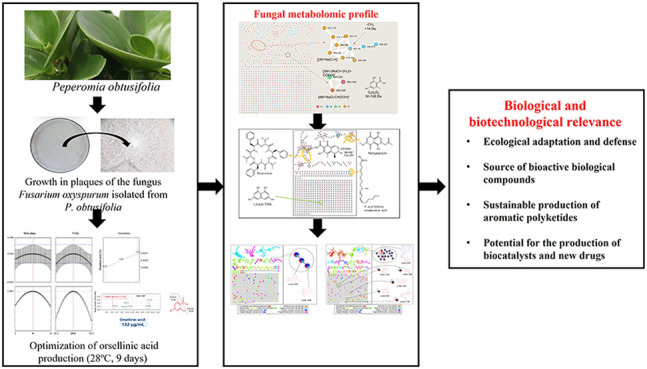

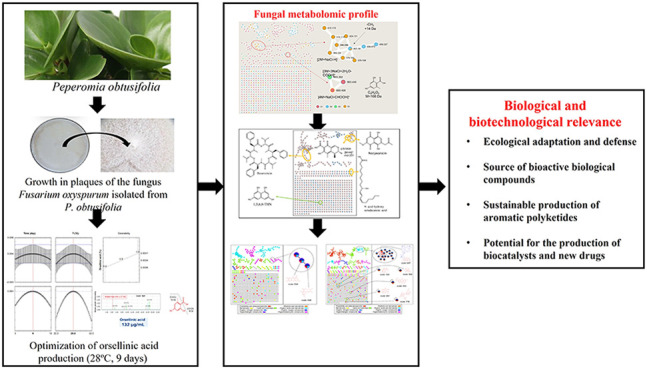

## 1 Introduction

The symbiotic association between plants and endophytic microorganisms plays a central role in plant adaptation, metabolism, and protection mechanisms. In this context, endophytes are microorganisms that colonize the internal tissues of plants without causing visible disease symptoms and establish highly dynamic biochemical interactions with their hosts.^1–4^ These interactions can directly influence plant physiology, promoting growth, increasing tolerance to abiotic and biotic stresses, modulating nutrient acquisition, and altering the biosynthesis of specialized metabolites.^5–8^ In many cases, endophytic fungi and bacteria act as metabolic partners, providing bioactive intermediates, signaling molecules, or complete biosynthetic pathways that expand the host plant’s chemical diversity.^9–14^

For example, recent studies have shown that endophytes can produce a wide range of secondary metabolites, including alkaloids, terpenoids, phenolic compounds, peptides, and aromatic polyketides, many of which exhibit relevant pharmaceutical and industrial properties.^3,4,15–17^ These metabolites are frequently involved in ecological communication processes, defense mechanisms, and adaptive responses that contribute to host survival under environmental stress.^5,6^ Furthermore, endophyte-mediated metabolic modulation can directly influence the accumulation of specialized metabolites in plants, reinforcing the concept of metabolic cooperation in plant-microbe symbioses,^7,8,12–14,18^

Among microbial metabolites, aromatic polyketides constitute one of the most structurally diverse and biologically relevant classes of natural products.^19–21^ Their biosynthesis is mainly associated with fungal polyketide synthases (PKSs), which generate highly reactive aromatic structures that can undergo extensive structural diversification.^22^ In this context, orsellinic acid is considered a central intermediate in the metabolism of fungal aromatic polyketides. It has been described as a precursor of several meroterpenoids, chromanes, benzopyrans, and depsides produced by fungi and lichens.^21–23^ In addition to their ecological relevance, these compounds exhibit antioxidant, antimicrobial, cytotoxic, and anti-inflammatory activities, highlighting their biotechnological potential.^15,21^

In this context, *Peperomia obtusifolia* has attracted increasing interest in phytochemistry due to the presence of prenylated chromanes and benzopyran derivatives with reported biological activities, including antioxidant, antimicrobial, cytotoxic, and anti-inflammatory effects. Furthermore, species of the genus *Peperomia* are widely used in traditional medicine for the treatment of inflammatory disorders, infections, and gastrointestinal diseases, which reinforces their relevance as a source of bioactive natural products. Structurally, prenylated chromanes are particularly interesting because prenylation increases lipophilicity and often improves membrane interaction, biological activity, and molecular stability of aromatic metabolites.^24–26^ These characteristics make prenylated meroterpenoids promising targets for pharmaceutical and biotechnological applications.

Therefore, the interaction between *Peperomia obtusifolia* and its endophytic microbiota represents a promising model for investigating metabolic cooperation between plants and fungi. For example, previous phytochemical studies have shown that *P. obtusifolia* accumulates prenylated chromanes and benzopyrans structurally related to aromatic polyketides,^10,11,15^ Proteomic and transcriptomic investigations have identified enzymes associated with meroterpenoid biosynthesis, including prenyltransferases and cyclases, but have failed to identify polyketide synthases directly associated with orselinic acid biosynthesis.^7,16,17^ This observation has raised the hypothesis that the aromatic precursors involved in chromane biosynthesis could originate from endophytic fungi associated with the plant. Supporting this hypothesis, previous metabolomic investigations of endophytic fungi associated with plants have demonstrated metabolic exchanges and biosynthetic complementarity between host plants and endophytic fungi.^8,18,27^ In particular, studies involving *Fusarium oxysporum* and *Diaporthe infecunda*, isolated from *P. obtusifolia*, have revealed the production of aromatic polyketides derived from the orsellinic acid pathway, including bikaverin, islandicin, lecanoric acid, and 6-methylsalicylic acid.^27^ These studies suggest that fungal-derived metabolites can act as biosynthetic intermediates in the formation of chromane derivatives accumulated in the host plant.

Therefore, this study investigates the role of the endophytic fungus *Fusarium oxysporum* Po18 in the production of aromatic polyketides associated with the specialized metabolism of *Peperomia obtusifolia*. Through culture optimization, LC-MS/MS metabolomics, and molecular network analyses, this work provides metabolomic evidence for possible metabolic cooperation between the fungus and its host plant, contributing to the understanding of fungus-plant metabolic interactions and the biosynthetic origin of plant-associated meroterpenoids. Optimization of fungal culture conditions was used to increase the accumulation of biosynthetic intermediates potentially involved in the formation of prenylated chromanes, as previously described in *P. obtusifolia*.

## 2 Materials and Methods

The experimental workflow was structured in a sequential and integrated manner to align with the study’s objectives. Initially, the cultivation parameters of *F. oxysporum* were optimized through a Central Composite Rotatable Design (CCRD) to maximize orsellinic acid production. HPLC-DAD was used to quantitatively analyze extracts obtained under optimal conditions, which were subsequently subjected to comprehensive metabolomic profiling using HPLC–ESI–qTOF–MS/MS. The resulting spectra were processed using the GNPS platform and MZmine to generate molecular networks and identify polyketide metabolites. This multistep strategy enabled the enrichment and identification of fungal aromatic polyketides potentially associated with the biosynthesis of prenylated chromanes previously reported in *P. obtusifolia*.

### 2.1 Reagents and Standards

Analytical-grade ethyl acetate was purchased from Labsynth (Brazil). HPLC-grade methanol was purchased from Merck (Germany), and HPLC-grade acetonitrile and formic acid were purchased from Sigma–Aldrich (USA). Standard orsellinic acid was purchased by Acros Organics (Belgium), and potato dextrose agar (PDA) from Neogen (Brazil)

### 2.2 Fungus Cultivation and Extraction of Metabolites

The *Fusarium oxysporum* strain Po 18, previously isolated from healthy leaves of *Peperomia obtusifolia* (GenBank accession number MH923264),^20^ was cultivated on potato dextrose agar in sterilized acrylic Petri dishes. Cultures were incubated at 28 °C in the dark for 7 days, and their purity was confirmed by morphological evaluation.

Afterward, *F. oxysporum* extracts were obtained using the method described by Jørgensen et al. (2014).^15^ The mycelium of the fungus was transferred to Erlenmeyer flasks containing ethyl acetate, which were stirred at 20 °C for 24 h. Subsequently, the organic fraction was separated, and the residue was subjected to two additional extractions. The pooled organic fraction was evaporated under reduced pressure, transferred to amber flasks, and stored at 4 °C. Ethyl acetate was selected due to its well-established efficiency in recovering weakly acidic and aromatic fungal polyketides, including orsellinic acid and its derivatives. The crude extracts were resuspended in methanol (1 mg/mL) and subjected to solid-phase extraction (SPE) using a Chromabond C-18 cartridge. The SPE was activated using 1.0 mL of a water/methanol (95:5) solution. Subsequently, 1.0 mL of the extract solution and 2.0 mL of methanol were added for complete elution.

The determination of orsellinic acid in the extracts was performed using a Shimadzu Prominence LC-20AD chromatograph (Japan)­equipped with LC-20AD pumps, DGU-20A3 degasser, SIL-20A autosampler, SPD-M20A photodiode-array detector, CTO-20A column oven, and CBM-20A controller. The samples were analyzed using a C_18_ column, Luna 5 μm C18(2) 100 Å, Phenomenex (UK), with 150 mm × 4.6 mm. The mobile phase consisted of A (an aqueous formic acid solution, 0.1% in water) and B (formic acid in acetonitrile, 0.1%). The gradient separation method consisted of 20–23% B (10 min), 23–100% B (2 min), and 100% B (4 min).

All analyses were performed in triplicate at 254 nm with a flow rate of 1.0 mL/min and a sample injection volume of 5–10 μL, depending on the matrix effect of the extracts. The orsellinic acid content was estimated as a percentage of the extracted mass (w/w). *Fusarium oxysporum* was cultivated under optimized period and temperature conditions, and the optimized extracts (n = 3) were evaluated by the described HPLC-DAD method.

### 2.3 Optimization of Orsellinic Acid Production

The influence of cultivation time (X_1_) and temperature (X_2_) on orsellinic acid production was evaluated using a Central Composite Rotatable Design (CCRD) with two central points. Cultures were performed in triplicate, and orsellinic acid content was analyzed as described. Statistical analysis was conducted using Statistica 14.0.0.15 (TIBCO Software Inc., USA). The most significant factors were identified using a Pareto chart at a 95% confidence level. Optimal conditions were estimated using the desirability function, calculated as the geometric mean of the individual desirability values. Cultivation at the predicted optimal condition (9 days, 26.8 °C) was used to obtain extracts for subsequent metabolomic analysis.

### 2.4 Metabolomic Analysis by HPLC-ESI-qTOF-MS/MS

The MS and MS/MS spectra were obtained from a chromatographic system, Shimadzu liquid chromatograph (Japan), composed of two analytical pumps model LC-20AD, automatic injector SIL-20AHT, UV/vis detector SPD-20A, column oven CTO-20A, and controller CBM-20A. In addition, the column Phenomenex Luna PFP (2) 5 μm, 150 × 2 mm, was used during the analyses. The mobile phase consisted of A (an aqueous formic acid solution, 0.1% in water) and B (formic acid in acetonitrile, 0.1%). The flow rate was 0.4 mL/min, with an exploratory gradient of 2% B for 3 min, followed by a 29 min gradient from 2% to 100% B, then 100% B for 2 min.

The ESI-QTOF-MS/MS system used was a Bruker microToF-QII model, operating in both positive ESI (+) and negative ESI (−) modes. The RF2, 2RF, and hexapole RF funnels were programmed at 400, 200, and 200 Vpp, respectively. In the ESI-(+) mode, MS and MS/MS spectra were acquired with the following parameters: N_2_ gas for nebulization and drying at 4 bar and 8L. min^–1^, respectively, capillary energy of 4,500 V, 200 °C drying temperature, and collision and quadrupole energy at 12 and 6 eV. In the ESI-(−) mode, spectra of MS and MS/MS were acquired with the following parameters: N_2_ gas for nebulization and drying at 8 bar and 8L min^–1^, respectively, capillary energy of 3,500 V, 200 °C drying temperature, and collision and quadrupole energy at 10 and 8 eV. Data obtained from this analysis were used to carry out the metabolomic study.

### 2.5 Molecular Networking and Data Processing

The metabolomic profiles of *F. oxysporum* Po 18 cultured in the optimized condition were obtained from MS and MS/MS data in the RAW extension acquired by the MassLynx software after analysis by HPLC-ESI-qTOF-MS/MS. The raw files were converted into mzXML using a complete Windows package that converts vendor formats to a GNPS (Global Natural Product Social Molecular Networking)-compatible format (Supporting Information).

A molecular network was created using the online workflow (https://ccms-ucsd.github.io/GNPSDocumentation/) on the GNPS website (http://gnps.ucsd.edu). The data were filtered by removing all MS/MS fragment ions within ±17 Da of the precursor *m*/*z*. MS/MS spectra were window-filtered to retain only the top 6 fragment ions within a ± 50 Da window throughout the spectrum. The precursor ion mass tolerance was set to 2.0 Da, and an MS/MS fragment ion tolerance of 0.5 Da. A network was then created, with edges filtered to have a cosine score above 0.7 and more than 4 matched peaks. Further, edges between two nodes were kept in the network if and only if each node appeared in the other’s top 10 most similar nodes.

The maximum molecular family size was set to 100, and the lowest-scoring edges were removed from molecular families until the molecular family size fell below this threshold. The spectra in the network were then searched against GNPS’ spectral libraries. The library spectra were filtered in the same manner as the input data. All matches kept between network spectra and library spectra were required to have a score above 0.7 and at least 4 matched peaks. Metabolite annotations were obtained through GNPS spectral library matching, combined with molecular networks and manual inspection of MS/MS fragmentation patterns. Cosine similarity scores were used throughout the GNPS workflow.

A network was established in which edges were selected based on a criterion requiring a cosine score exceeding 0.7 and at least 4 coincident peaks. The edges between the two nodes were kept in the network only if each appeared in the top 10 most similar aspects. Subsequently, the network spectra were searched against GNPS spectral libraries. The data were incorporated into the network using the GNPS MolNetEnhancer workflow (https://ccms-ucsd.github.io/GNPSDocumentation/molnetenhancer/) on the GNPS website (http://gnps.ucsd.edu).^27^ In addition, MS/MS data were processed using the MZmine software to get more annotations of substances by searching the KEGG database.^17,21–23^

## 3 Results and Discussion

Several questions remain regarding the molecular interactions and coevolution between plants and fungi. In this context, many studies have sought to understand the correlation between fungal aromatic polyketide production and plant symbiosis.^27–29^ In this context, the cultivation conditions of *F. oxysporum* Po 18 were initially optimized for orsellinic acid production, thereby avoiding its biotransformation into downstream products of the metabolic pathway. The extracts obtained under all cultivation conditions (Table 1) were initially analyzed and divided into groups based on the methodologies used to generate molecular networks.

**Table 1:** Orsellinic Acid Content (% w/w) in *Fusarium oxysporum* Po 18 Extracts under Different Cultivation Time and Temperature Conditions, as Determined by HPLC-DAD^a^

Time/X_1_ (days)	Temperature/X_2_ (°C)	Orsellinic acid (%)
4.8 (−1)	23.5 (−1)	0.0007 ± 0.0001
4.8 (−1)	30.5 (+1)	0.0008 ± 0.0001
13.2 (+1)	23.5 (−1)	0.0039 ± 0.0005
13.2 (+1)	30.5 (+1)	0.0012 ± 0.0002
9.0 (0)	27.0 (0)	0.0040 ± 0.0001
9.0 (0)	27.0 (0)	0.0041 ± 0.0001
9.0 (0)	22.0 (−1.45)	0.0010 ± 0.0001
9.0 (0)	32.0 (+1.45)	0.0026 ± 0.0001
3.0 (−1.45)	27.0 (0)	0.0036 ± 0.0005
15.0 (+1.45)	27.0 (0)	0.0027 ± 0.0003

a The coded values (−1, 0, +1, and ±1.45) correspond to the levels of the Central Composite Rotatable Design (CCRD), where −1 and +1 represent the low and high factorial levels, 0 represents the central point, and ±1.45 correspond to the axial points used to estimate quadratic effects.

Data analysis showed that temperature and cultivation time significantly influenced orsellinic acid production by *Fusarium oxysporum* Po18. Figure 1A presents the Pareto chart, indicating that temperature was the most significant variable affecting metabolite accumulation, followed by cultivation time. The response surface (Figure 1B) shows that orsellinic acid production was maximized at intermediate temperatures and with longer cultivation periods, suggesting that extreme temperatures negatively affect the fungus’s secondary metabolism. Figure 2 illustrates the desirability analysis used to predict the ideal cultivation conditions for maximizing orsellinic acid accumulation. The combined evaluation of cultivation time and temperature indicated that the highest desirability value was obtained at approximately 9 days and 26.8 °C, conditions subsequently selected for metabolomic analyses.

**Figure 1:**
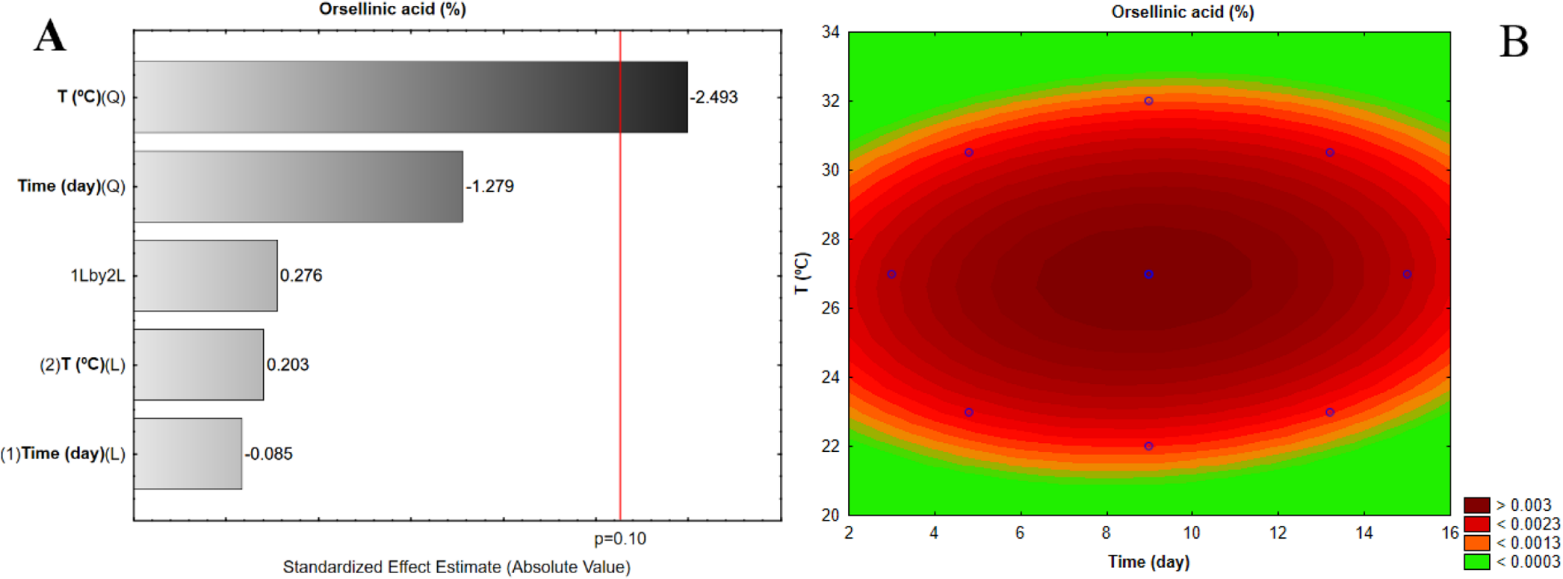
Statistical analysis of the influence of cultivation parameters on orsellinic acid production: (A) Pareto chart showing the significance of time and temperature (linear and quadratic terms) on the metabolite yield; (B) Contour plot illustrating the response surface of orsellinic acid content as a function of cultivation time (X_1_) and temperature (X_2_).

**Figure 2:**
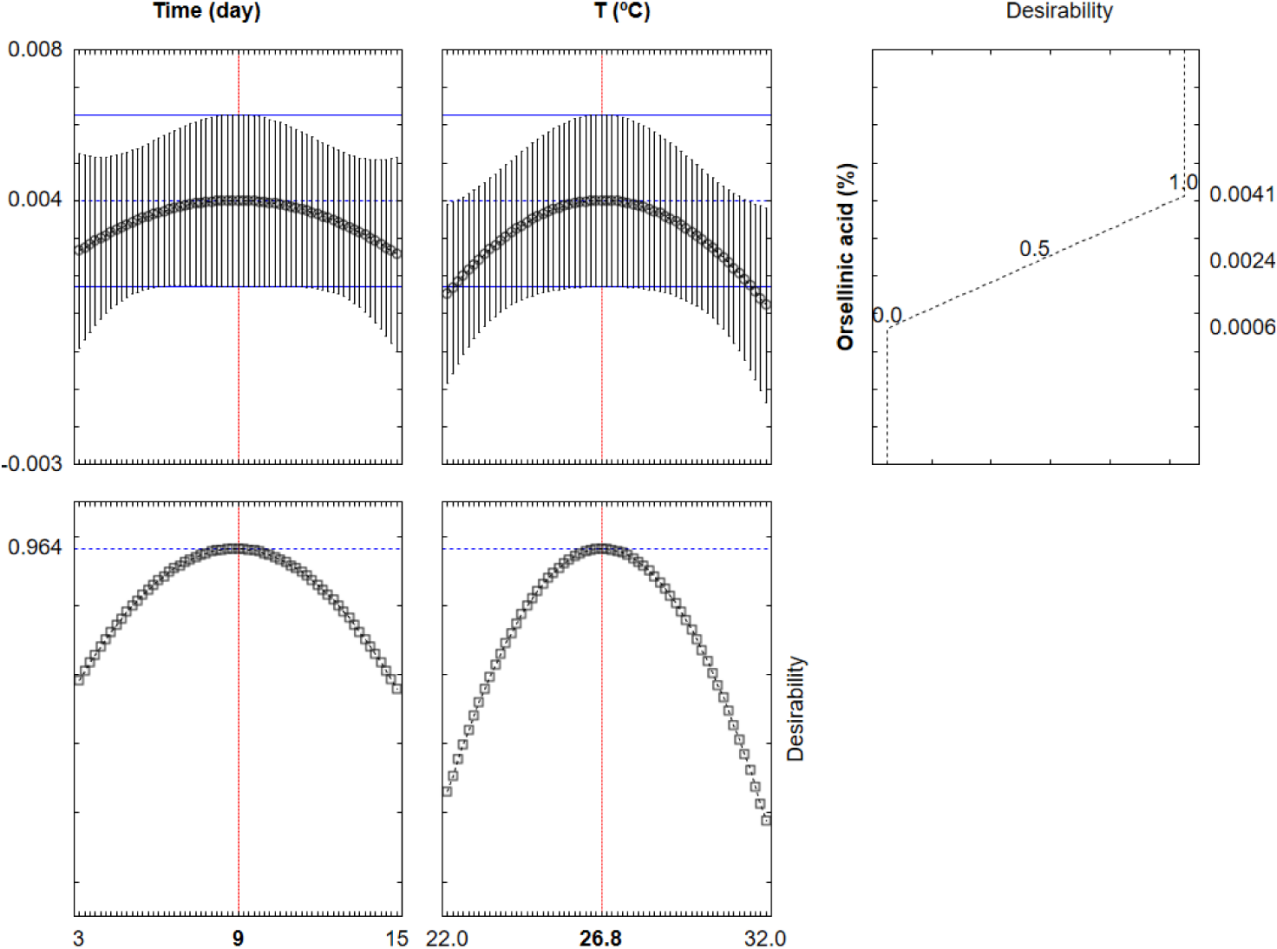
Optimization of cultivation conditions for enhanced orsellinic acid production by *Fusarium oxysporum* Po 18.

The condition optimized to produce orsellinic acid was compared with the standard condition to obtain clusters that yielded MS and MS/MS spectra with fragmentation patterns identical to or similar to that of this compound. After validating the data and performing UPLC-MS/MS analysis of the extract in ESI-(−) mode to conduct metabolomic studies, the data obtained were separated into four groups: crude extract (G1 and G2) and orsellinic acid standard (G3 and G4). The red and blue nodes were generated from the grouping of MS spectra of the ESI-(+) mode, categorized in the G1 group, and ESI-(−) mode spectra in the G2 group. The green and yellow nodes generated 72 nodes from the grouping of MS spectra from the ESI-(+) mode (G3 group) and the ESI-(−) mode (G4 group) (Figure 3).

**Figure 3:**
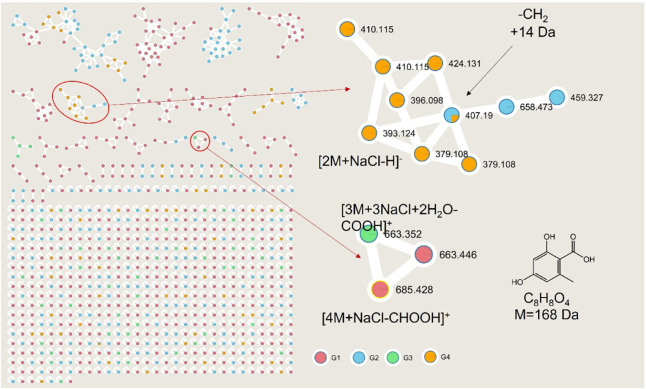
Molecular network obtained from MS/MS data of *Fusarium oxysporum* Po 18 extracts cultured under optimized conditions. *Highlighted nodes represent precursor ions with fragmentation patterns like orsellinic acid, annotated based on GNPS spectral library. Red and blue nodes indicate data acquired in positive- and negative-ionization modes, respectively—network generated using GNPS default parameters for qualitative visualization of structural relationships.

A metabolomic study aimed to detect orsellinic acid and related biosynthetic metabolites, including polyketides, to understand the interaction between the endophyte *F. oxysporum* Po 18 and its host plant *P. obtusifolia*. Therefore, the UPLC-MS/MS data generated for the metabolomic study were compared with established references using the GNPS platform. This comprehensive investigation elucidated the congruence between the experimental results and the proposed fragmentation mechanisms of orsellinic acid, confirming its production in *F. oxysporum*. Evidence for its identification was provided by detecting fragment ion [M-H-44]^−^ at *m*/*z* 167.0329→123.0422 resulting from the decarboxylation of orsellinic acid, with the initial negative charge on the hydroxyl group. Additionally, the initial negative charge on the COOH group allowed the fragmentation path *m*/*z* 123.0422→81.0333, resulting from the loss of C_2_H_2_O (Supporting Information S1 and S2).^30^ An orsellinic acid standard was used during the analysis.

Subsequently, groups G3 and G4 were inhibited from performing more comprehensive MS data processing, available through the GNPS platform, to visualize MS classes and substances related to the metabolic dynamics of *F. oxysporum* divided into alkaloids, aromatic derivatives, lipids, nucleotides, organic acids, nitrogenous derivatives, oxygenated derivatives, heterocyclic derivatives, phenylpropanoids, and polyketides (Figure 4) were noted (Supporting Information S3 and S4).

**Figure 4:**
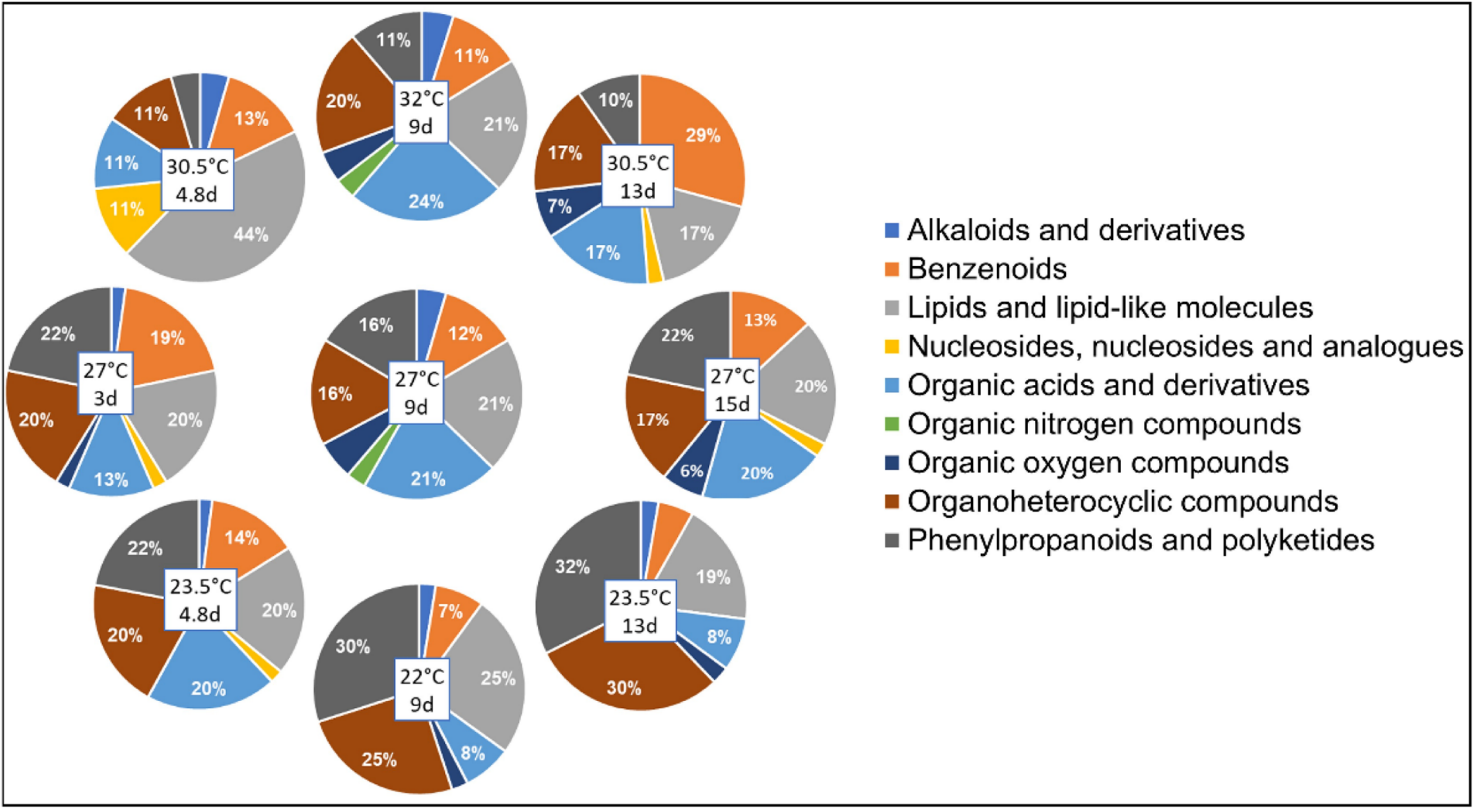
Metabolite class distribution in *Fusarium oxysporum* Po 18 extracts under different cultivation conditions: Classification obtained using the MolNetEnhancer and NAP workflows on the GNPS platform. Results highlight major compound classes, including polyketides, phenylpropanoids, lipids, and aromatic derivatives.

After optimizing fungal growth, it was possible to establish a condition that favored orsellinic acid accumulation, enabling the metabolomic study to be performed under this condition. Therefore, after analyzing the data using the GNPS library, it was possible to identify several nodes as similar or identical to the spectra of beauvericin, 9-hydroxyoctadecadienoic acid, 1,3,6,8-tetrahydroxynaphthalene (1,3,6,8-THN), citrinin, and norjavanicin (Figure 5). Optimizing fungal cultivation conditions was essential to increase the accumulation of aromatic PKS-derived metabolites, enabling the detection of biosynthetic intermediates structurally compatible with prenylated chromanes previously described in *P. obtusifolia*. This approach enabled the investigation of a possible metabolic interaction between the endophytic fungus and the host plant.

**Figure 5:**
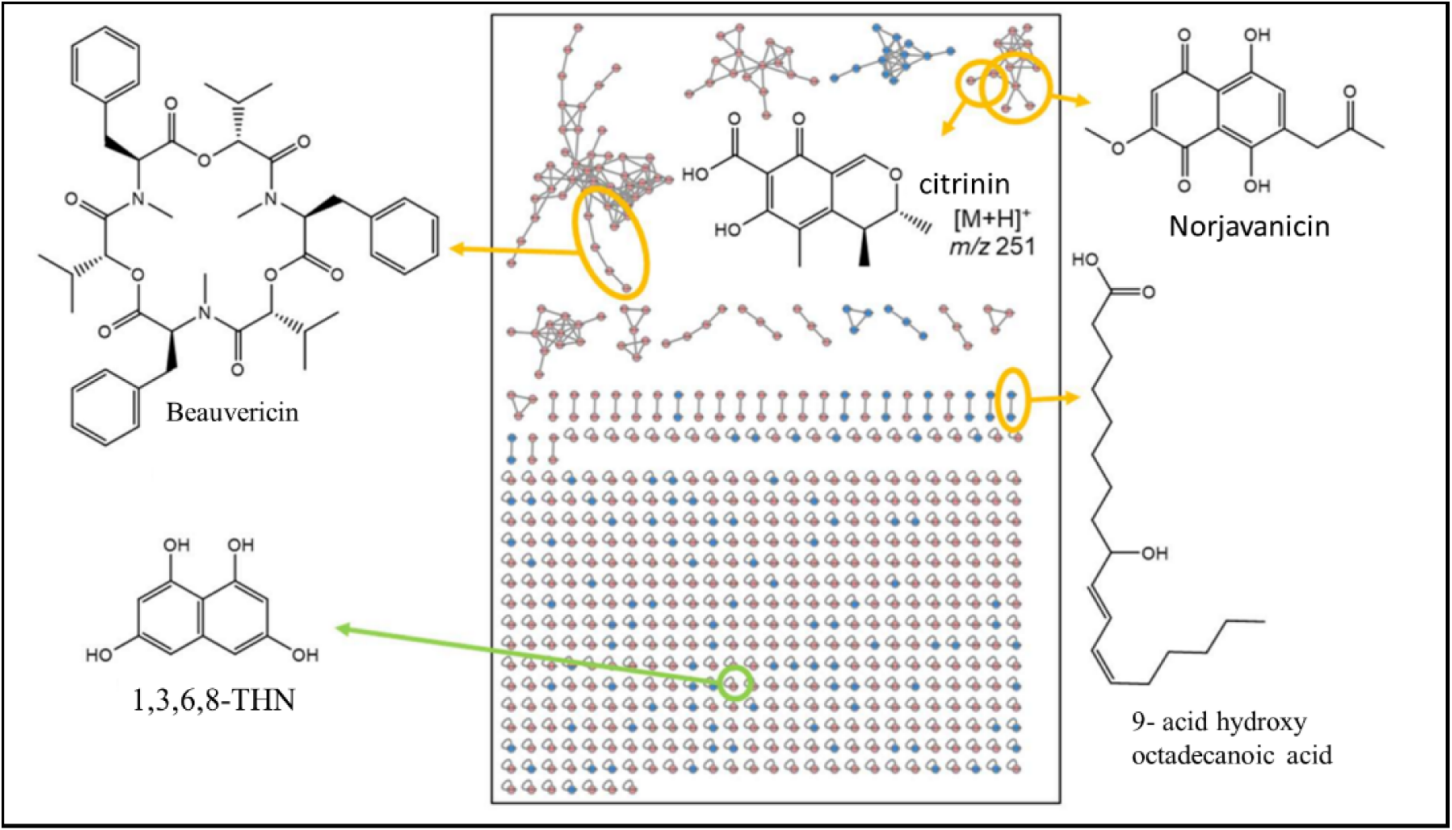
Annotated molecular interaction network of *Fusarium oxysporum* Po 18 metabolites obtained under optimized culture conditions: Nodes represent precursor ions; red and blue indicate positive and negative ionization modes, respectively. Clusters include metabolites such as beauvericin, 1,3,6,8-tetrahydroxynaphthalene, and citrinin, annotated based on GNPS and UNIFI spectral databases. *Gray nodes represent features that could not be annotated due to the absence of corresponding reference spectra in public libraries, low signal intensity, or insufficient quality of MS/MS fragmentation.

Optimizing fungal cultivation conditions was essential to increase the accumulation of aromatic metabolites derived from PKS, thereby enabling the detection of biosynthetic intermediates structurally compatible with prenylated chromanes previously reported in *Peperomia obtusifolia*. This study allowed the investigation of a possible metabolic interaction between the endophytic fungus and the host plant. Therefore, the biosynthetic origin of the prenylated chromanes detected in *P. obtusifolia* was probably heterologous, involving cooperation between the plant and its endophytic fungus. The identification of orselinic acid, lecanoric acid, and related polyketides in extracts of *F. oxysporum*, all products of the fungal polyketide synthase pathway, corroborates the hypothesis that aromatic precursors are synthesized by the fungus and subsequently prenylated or cyclized by the plant’s enzymatic machinery. This metabolic complementarity aligns with the concept of “biosynthetic partnership,” in which endophytes provide polyketide structures that the host plant subsequently transforms into complex meroterpenoids.

Previous studies on *Peperomia obtusifolia* reported prenylated chromanes and benzopyran derivatives potentially associated with the orsellinic acid biosynthetic pathway; however, aromatic polyketide intermediates such as orsellinic acid itself had not been directly detected in the plant. This discrepancy can be attributed to differences in physiological states, environmental conditions, or the absence of active endophytic colonization at the time of sampling. Under controlled conditions, the optimized cultivation of *F. oxysporum* Po18 enabled the accumulation and detection of low-abundance fungal polyketides that may not be detectable in plant extracts alone. Therefore, this work expands the known chemical diversity of *P. obtusifolia* by revealing previously hidden fungal-derived intermediates involved in chroman biosynthesis.

A subsequent investigation was directed to explore downstream products, including chromanes in *P. obtusifolia*. By examining the presence of these aromatic polyketides, it was suggested that changes in orsellinic acid concentration in the samples could be linked to the further conversion of these polyketides, resulting in lower orsellinic acid accumulation in *F. oxysporum*. Therefore, data analysis using the molecular network enabled the identification of clusters with spectra similar to G1 with G3 and G2 with G4; however, without annotations based on the library available on the GNPS platform. All HPLC-MS data from *F. oxysporum* were ungrouped and reanalyzed individually to examine metabolic variation and annotate aromatic polyketides. It was possible to observe that the range of temperature and the time course of cultivation influenced the metabolism of *F. oxysporum* at each sampling condition. Therefore, several anthraquinones, commonly found in Fusarium species, corroborate the work of research groups (Table 2).^31,32^

**Table 2:** Metabolites Identified in the Molecular Network Based on GNPS Spectral Matching, Including Molecular Ions and Theoretical Exact Mass

Compound	Retention time (min)	Molecular ion	Exact mass (Da)	Detected ion (*m*/*z*)
citrinin	2.26	[C_13_H_14_O_5_+H]^+^	250.0841	251.0497
norjavanicin	4.88	[C_14_H_12_O_6_–C_2_H_2_O]^+^	276.0634	235.0579
9-hydroxy octadecadienoic acid	6.59	[C_18_H_32_O_3_–H]^−^	296.2355	295.155
1,3,6,8-THN	7.01	[C_10_H_8_O_4_–H]^−^	191.0422	191.033
beauvericin	17.10	[C_45_H_57_N_3_O_9_+H]^+^	783.4095	784.4182
beauvericin	17.10	[C_45_H_57_N_3_O_9_+NH_4_]^+^	783.4095	801.4119

Spectral data from the ESI-(+) mode revealed similarity patterns (cosine >0.7) between the MS/MS spectra from the GNPS platform database and the experimental results. Among the similarities in the MS data, some results showed that PKSs biosynthesize aromatic polyketides with architectures similar to those involved in the orsellinic acid biosynthetic pathway.^33,34^ Furthermore, through MZmine analysis, additional polyketides, including bicaverine, 3-linalylflaviolin, scytalone, and lecanoric acid, among other were identified in the metabolomic study (Table 3),^15,23,27^ which corroborates previous studies^11,16,17,35–37^

**Table 3:** Aromatic Polyketides Detected in *Fusarium oxysporum* Po 18 Extracts Based on MZmine-Processed MS/MS Data in Positive and Negative Ionization Modes

Compound	Retention time (min)	Molecular ion	Detected ion (*m*/*z*)
3-hydroxyjuglone	2.8	[C_10_H_6_O_4_+H]^+^	191.034
cyperine	13.1	[C_15_H_16_O_4_+H]^+^	261.112
lecanoric acid	10.8	[C_16_H_14_O_7_+H]^+^	319.080
6-methylsalicylate	13.5	[C_8_H_8_O_3_–H]^−^	151.040
1-naphthol	14.1	[C_10_H_8_O–H]^−^	143.050
scytalone	14.1	[C_10_H_10_O_4_+H]^+^	195.065
β-zearalenol	14.4	[C_18_H_24_O_5_+H]^+^	321.169
bikaverin	16.6	[C_20_H_14_O_8_+Na]^+^	405.056
zearalenone	16.9	[C_18_H_22_O_5_+H]^+^	319.154
zearalanol	17.3	[C_18_H_26_O_5_+Na]^+^ [C_18_H_26_O_5_–H]^−^	345.167
3-linalylflaviolin	18.1	[C_20_H_22_O_5_–H]^−^	341.140
α-zearalenol or zearalanone	19.1	[C_18_H_24_O_5_+H]^+^	321.169
		[C_18_H_24_O_5_–H]^−^	319.169
3,4-dehydro-6-hydroxymellein	19.7	[C_10_H_8_O_4_–H]^−^	191.035

The biosynthetic profile observed for *F. oxysporum* Po18 is consistent with the activity of iterative type I PKSs that generate tetraketide intermediates, subsequently cyclized to form orsellinic acid. The MS/MS fragmentation sequence (*m*/*z* 167.0329 → 123.0422 → 81.0333) supports this mechanism, reflecting decarboxylation and characteristic losses of C_2_H_2_O associated with ortho-hydroxylated aromatic polyketides. Once produced by the fungus, orsellinic acid acts as a chemically activated precursor for plant tailoring reactions. Previous proteomic and transcriptomic analyses of *P. obtusifolia* indicate the presence of prenyltransferases, oxidases, and cyclases capable of converting simple polyketide acids into prenylated chromans. Therefore, the metabolomic evidence supports a cooperative biosynthetic model in which the fungus provides the aromatic scaffold, while the plant performs downstream modifications leading to the meroterpenoids previously reported in *P. obtusifolia*.

It is also important to highlight the potential biosynthetic gene clusters (BGCs) in *Fusarium* species that may underlie the observed metabolic profiles. For example, previous studies have reported that *Fusarium* genomes harbor multiple type I iterative polyketide synthase (PKS) clusters, responsible for the biosynthesis of aromatic polyketides, including bikaverin, citrinin, and related metabolites. The detection of such compounds in this study corroborates the activity of these PKS systems, suggesting that the same or closely associated clusters may also participate in the production of orsellinic acid and its derivatives, as well as conserved fungal polyketide pathways.^15,23^

In this context, it was possible to establish, for the first time, a direct metabolic link between *Fusarium oxysporum* Po 18 and *Peperomia obtusifolia*. The identification of orsellinic acid was confirmed by LC-MS/MS analysis and comparison with an authentic standard, thereby ensuring structural reliability. Metabolomic analysis, integrating molecular networks and spectral library matching, provided a comprehensive view of the fungal contribution to the biosynthesis of chroman-bearing meroterpenoids in the host plant, demonstrating the symbiotic role of endophytic fungi and highlighting their potential for supplying metabolites for the biosynthesis of natural products.

## 4 Conclusion

This study provides the first experimental evidence that the endophytic fungus *Fusarium oxysporum* Po18 actively contributes to the biosynthesis of aromatic polyketides in *Peperomia obtusifolia*. Metabolomic profiling combined with molecular networking confirmed the production of orsellinic acid and several related intermediates, revealing a fungal origin for key precursors of meroterpenoids in the host plant.

Optimization of cultivation parameters significantly enhanced orsellinic acid yield, demonstrating the potential of *F. oxysporum* as a sustainable biotechnological source of high-value aromatic polyketides. Beyond its chemotaxonomic relevance, this discovery establishes a model for exploring metabolic cooperation in plant–endophyte systems. The integrated use of LC–MS/MS metabolomics and molecular networking has proven to be a powerful strategy for uncovering hidden fungal biosynthetic pathways, paving the way for future studies aimed at engineering endophytic fungi for natural product discovery and production.

## Supplementary Material



## Notes

GNPS molecular networking data sets for ESI­(+) and ESI(−) modes; chromatographic profile of the extracted ion (m/z 167.0356) corresponding to the orsellinic acid annotation and MS/MS spectrum (Figure S1); proposed fragmentation mechanisms of orsellinic acid in the ESI(−)-MS/MS mode (Figure S2); categorization of metabolite classes in the molecular networking matrix and annotation of polyketides based on clusters processed from ESI­(+)-MS/MS data (Figure S3); categorization of metabolite classes in the molecular networking matrix and annotation of polyketides based on clusters processed from ESI(−)-MS/MS data (Figure S4) (PDF)
