# Supplementary material for: Orsellinic Acid from the Endophytic Fusarium oxysporum Drives Specialized Metabolism in Peperomia obtusifolia
Source: ACS Omega. 2026 Jul 15;11(29):43107–15. doi: 10.1021/acsomega.5c12178 (PMC13425474; doi:10.1021/acsomega.5c12178)
Supplement: Supplementary file 1 [file ao5c12178_si_001.pdf]

Orsellinic Acid from the Endophytic *Fusarium oxysporum* Drives Specialized Metabolism in *Peperomia obtusifolia*

Wellington Gomes de Lima[1], Andreia de Araújo Morandim-Giannetti[2], João Luiz Bronzel Júnior[1], Silvia Noelí López[3], Massuo Jorge Kato[4], Maysa Furlan[1*]*

[1] Institute of Chemistry, Universidade Estadual Paulista (UNESP), Araraquara-SP, Brazil

[2] Department of Chemical Engineering, Centro Universitário da FEI, São Bernardo do Campo - SP, Brazil

[3] Pharmacognosy, Faculty of Biochemical and Pharmaceutical Sciences, Universidad Nacional de Rosario - CONICET, Rosario, Argentina

[4] Institute of Chemistry, Universidade de São Paulo (USP), São Paulo – SP, Brazil

Supplementary Materials

The molecular network links used in this work:

ESI(+) mode:

https://gnps.ucsd.edu/ProteoSAFe/status.jsp?task=b85299596e9e49d9ad3648fc7cc4eb15

SEI(+) mode:

https://gnps.ucsd.edu/ProteoSAFe/status.jsp?task=84619fcc64d94eac866763ea071ddef9

Fig. S1 presents the mass data of orsellinic acid, and Fig. S2 presents proposed fragmentation mechanisms of orsellinic acid in ESI-(-) mode.

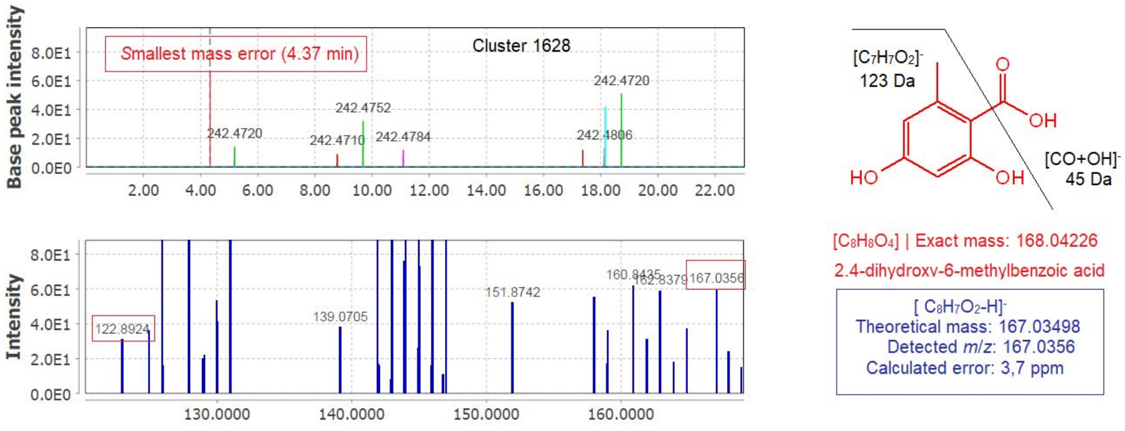

Figure S1. Chromatographic profile of the extracted ion (*m*/*z* 167.0356) for the corresponding 1628 cluster, the orsellinic acid annotation, and spectrum MS/MS with parent mass (cosine=0.80).

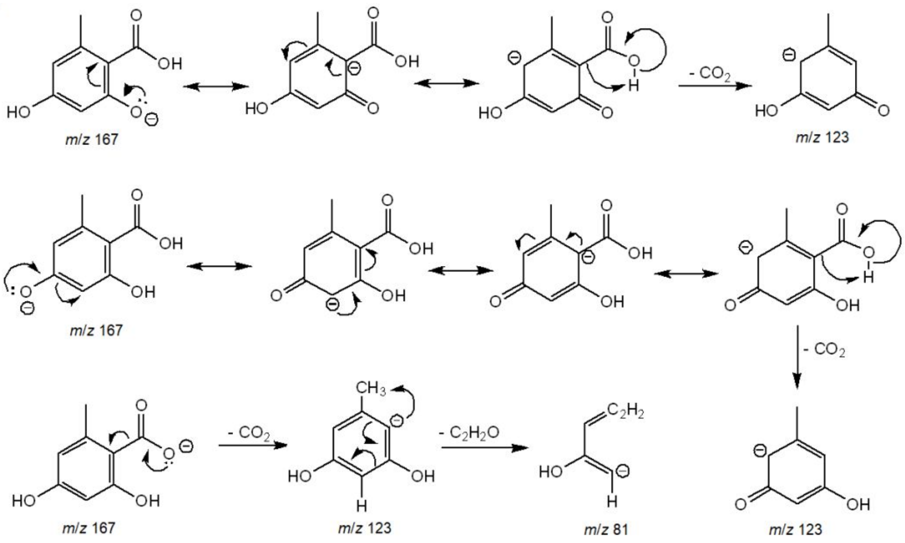

Figure S2. Proposal of orsellinic acid fragmentation mechanisms in the ESI-(-) mode by MS/MS of *m/z* 167;0356.

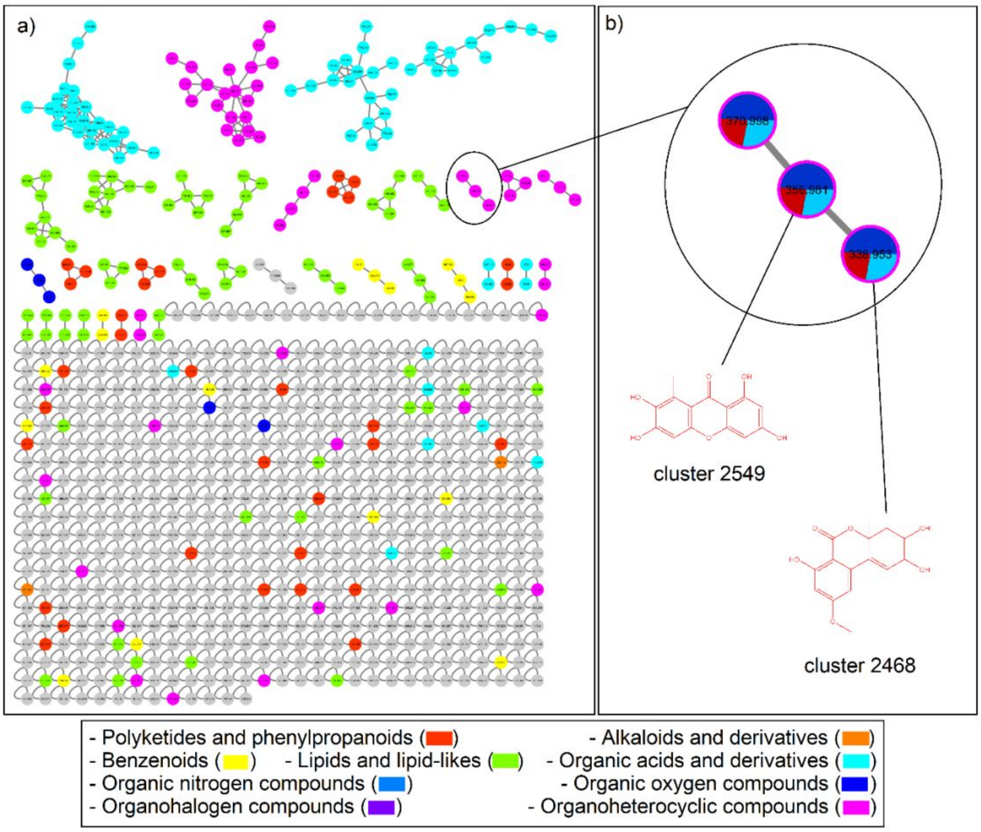

Figure S3. Categorization of classes of compounds in the molecular network matrix (a) and annotation of polyketides based on the clusters processed from ESI-(+)-MS/MS positive mode (b).

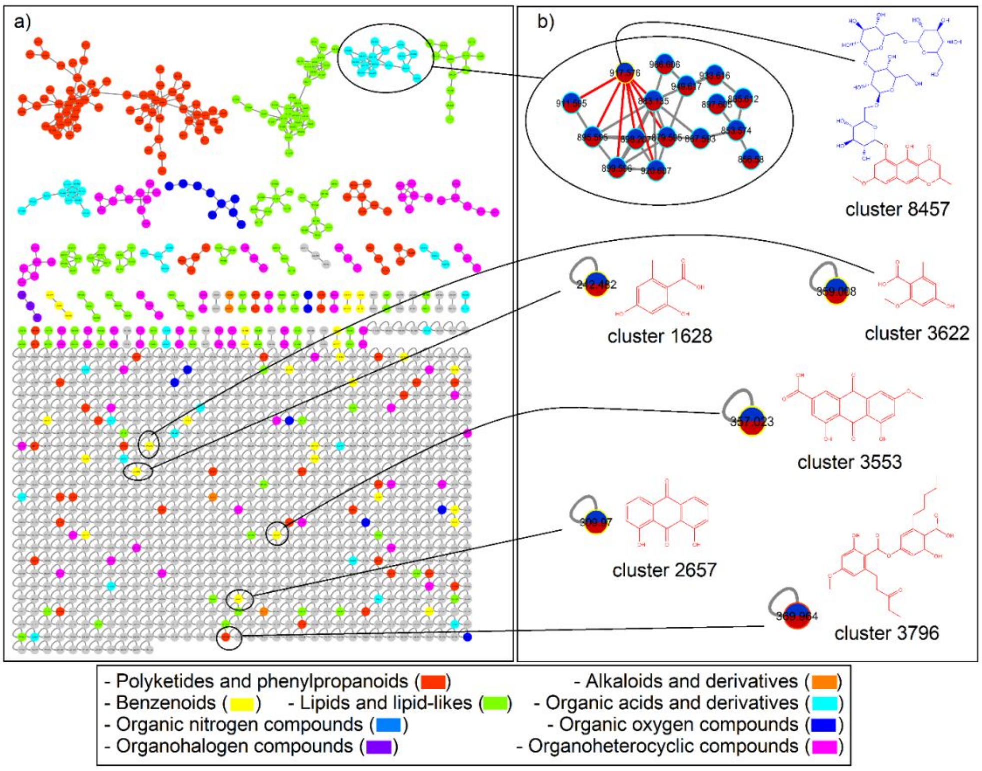

Figure S4. Categorization of classes of compounds in the molecular network matrix (a) and annotation of polyketides based on the clusters processed from ESI-MS/MS negative mode (b).
